# Rgp1 contributes to craniofacial cartilage development and Rab8a-mediated collagen II secretion

**DOI:** 10.3389/fendo.2023.1120420

**Published:** 2023-02-09

**Authors:** Dylan J. Ritter, Dharmendra Choudhary, Gokhan Unlu, Ela W. Knapik

**Affiliations:** ^1^ Department of Cell and Developmental Biology, Vanderbilt University, Nashville, TN, United States; ^2^ Division of Genetic Medicine, Department of Medicine, Vanderbilt University Medical Center, Nashville, TN, United States

**Keywords:** Rgp1, collagen, chondrocytes, zebrafish, cartilage, extracellular matrix

## Abstract

Rgp1 was previously identified as a component of a guanine nucleotide exchange factor (GEF) complex to activate Rab6a-mediated trafficking events in and around the Golgi. While the role of Rgp1 in protein trafficking has been examined *in vitro* and in yeast, the role of Rgp1 during vertebrate embryogenesis and protein trafficking *in vivo* is unknown. Using genetic, CRISPR-induced zebrafish mutants for Rgp1 loss-of-function, we found that Rgp1 is required for craniofacial cartilage development. Within live *rgp1^-/-^* craniofacial chondrocytes, we observed altered movements of Rab6a^+^ vesicular compartments, consistent with a conserved mechanism described *in vitro*. Using transmission electron microscopy (TEM) and immunofluorescence analyses, we show that Rgp1 plays a role in the secretion of collagen II, the most abundant protein in cartilage. Our overexpression experiments revealed that Rab8a is a part of the post-Golgi collagen II trafficking pathway. Following loss of Rgp1, chondrocytes activate an Arf4b-mediated stress response and subsequently respond with nuclear DNA fragmentation and cell death. We propose that an Rgp1-regulated Rab6a-Rab8a pathway directs secretion of ECM cargoes such as collagen II, a pathway that may also be utilized in other tissues where coordinated trafficking and secretion of collagens and other large cargoes is required for normal development and tissue function.

## Introduction

The extracellular matrix (ECM) is a complex network of proteins that provides architectural support for cellular and tissue development (1–3). Among ECM proteins, collagens are the most abundant in vertebrates, composing over a quarter of the dry body mass in humans (4, 5). In order for collagen to function, it must be trafficked from the site of synthesis, the endoplasmic reticulum (ER), to the Golgi complex before being secreted to the extracellular space (6–8). Previous studies have identified components of the secretory machinery contributing to collagen trafficking from the ER to the Golgi, including TANGO1, CREB3L2, and the Coat Proteins II (COPII) complex components Sec13-31, Sec23A, and Sec24D (9–17). Fewer studies addressed the role of the secretory machinery components in collagen transit through the Golgi complex (18, 19). Thus, this stretch of the collagen secretory pathway is less well understood. Consistently, studies in animal models, including zebrafish, have revealed that proper functioning secretory pathway is essential for craniofacial development (3, 20–22). Following initial mesenchymal condensations, chondroblasts are normally positioned and form individual cartilage elements (23–25). However, later steps of cartilage development depend on ECM secretion and isometric growth of cells. Chondroblasts at this stage are most affected by defects in the secretion of collagen and other cargoes to ECM, resulting in a thin matrix and clinically brittle skeletal elements (26).

Fibrillar collagens oligomerize extracellularly into cable-like fibrils and are indispensable for three-dimensional structural stability of cartilage and other tissues (27–29). Mutations in the collagen II α1 peptide, the predominant fibrillar collagen in cartilage, contribute to a category of diseases known as type II collagenopathies (30–34). In addition to mutations in the collagen II α1 peptide itself, mutations in its processing enzymes, including *LH2* and *LH3*, have also been found to cause developmental defects in skeletal tissues (9–11, 18, 35–38). Despite considerable knowledge of the early, ER-to-Golgi trafficking of fibrillar collagens, the mechanisms governing collagen transit from Golgi-to-plasma membrane remain relatively unknown (39–42).

The central regulators of the secretory pathway are small GTPases that need to be activated by selective GEFs (43). Like other Rabs, Rab6a is only active in intracellular traffic when it is bound to GTP and a cellular membrane following interactions with its cognate heterodimer GEF composed of Rgp1 and Ric1 (43–49). When bound to GDP, Rabs are inactive, cytosolic proteins and do not participate in trafficking processes. Previous biochemical studies and cell culture experiments identified the obligatory nature of this GEF complex and its role in activation of Rab6a (44, 45). The UniProt database associates RGP1 with ECM organization and vesicular transport (50). However, Mendelian diseases were not linked to *RGP1* deficiency and the impact on clinical phenome was only tested as part of genome wide analyses. Thus, little is known about the role of *RGP1* in clinical and developmental settings.

To address the biological function of Rgp1, we generated CRISPR-edited zebrafish knockout models and found that mutants have abnormal craniofacial cartilage development and defective collagen II secretion from chondrocytes. Furthermore, we show that Rab6a and Rab8a are critical contributors to the Rgp1-dependent secretion of collagen II in chondrocytes. Specifically, our results suggest that Rgp1, Rab6a, and Rab8a coordinate the traffic of collagen II to the extracellular space as part of a post-Golgi Rab pathway. We expect that our *in vivo* findings at the cellular and tissue levels will contribute to a greater understanding of protein trafficking across tissues specialized for large cargo secretion.

## Materials and methods

### Fish maintenance and breeding

Zebrafish were raised under standard laboratory conditions at 28.5°C with a constant photoperiod (14 h light: 10 h dark) as previously described (18).

### CRISPR/Cas9 genome editing

CRISPR/Cas9 target sites within the zebrafish *rgp1* gene were identified using the CHOPCHOP web tool (51). sgRNA templates were generated as previously described (52). gRNAs were synthesized with the MEGAshortscript T7 transcription kit (AM1354, ThermoFisher Scientific; Waltham, MA).

**Figure 1:**
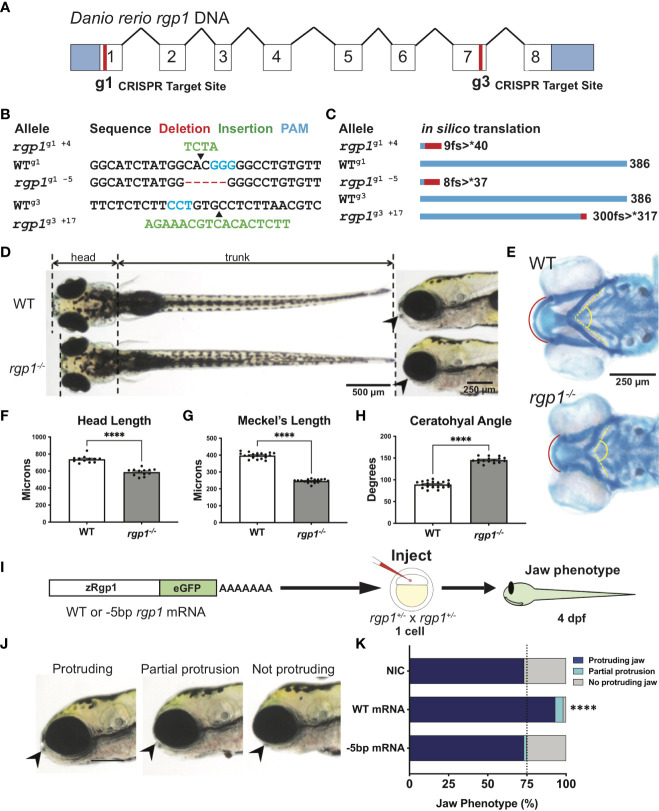
Rgp1 is required for zebrafish craniofacial skeletal development. **(A)** Zebrafish *rgp1* intron/exon map showing locations of g1 and g3 gRNA target sites. **(B)** Sequences from three different alleles isolated from the use of g1 and g3 gRNAs. **(C)** Predicted peptide lengths for alleles shown in **(B)**. **(D)** Live imaging of 5 dpf WT and *rgp1^-/-^* larvae from ventral and lateral view showing jaw protrusions (black arrowhead). **(E)** Alcian blue staining and imaging of 5 dpf WT and *rgp1^-/-^* cartilage in the ventral craniofacial head skeleton. The Meckel’s cartilage (red), and ceratohyal (yellow, dashed) are marked. **(F)** Quantification of larva head length. **(G)** Quantification of Meckel’s cartilage length. **(H)** Quantification of ceratohyal cartilage angle. **(I)** Schematic for zebrafish *rgp1* mRNA injection and rescue experiment. **(J)** Lateral images of larvae showing jaw protrusion, partial protrusion, and no protrusion phenotypes (black arrowheads). **(K)** Quantification of jaw phenotype percentages in groups of injected and non-injected embryos. Significance is presented by ****p<0.0001.

To generate mutations with CRISPR/Cas9 system, a mixture of 500 pg purified Cas9 protein (CP01, PNA Bio Inc.; Newbury Park, CA) and 150 pg gRNA was injected into one-cell stage WT AB embryos. Injected embryos were grown to 5 dpf for phenotypic analysis and to identify founders carrying mutant alleles in the germline. Founders were then outcrossed to transgenic or WT lines to generate stable lines carrying the listed mutations (**Figure 1B**). *In silico* protein translation was based on cDNA sequences obtained from mutant larvae (**Figure 1C**).

### Live imaging of larvae for body length analysis

Larvae were anesthetized at 5 dpf in 0.15 mg/mL Tricaine (TRS1, Pentair; Cary, NC) and mounted in 3% methylcellulose on a bridge slide. Body lengths were measured from jaw protrusion to caudal fin fold from dorsal views in FIJI software (53). Head lengths were measured from jaw protrusion to the pectoral fin attachment point.

### Cartilage staining

Larvae at 5 dpf were fixed in 2% PFA and stained overnight in 0.02% Alcian Blue (A5268, Sigma; St. Louis, MO) and 60 mM MgCl_2_ in 70% EtOH as previously described (54). Larvae were then bleached (1.5% H_2_O_2_, 1% KOH), washed in 0.25% KOH/25% glycerol, and cleared in 0.25% KOH/50% glycerol.

### RNA isolation and cDNA synthesis

RNA was isolated from at least 30 3 dpf larvae from independent mating crosses of fish. Larvae were homogenized in TRIzol Reagent (15596018, ThermoFisher Scientific; Waltham, MA), and RNA extraction was performed as previously described (55). RNA (1 µg) obtained from samples was treated with DNase I before inactivation by EDTA chelation. Samples were mixed with an 18-nucleotide poly-T primer and allowed to anneal before adding dNTPs and M-MLV Reverse Transcriptase (M1701, Promega; Madison, WI).

### mRNA overexpression and phenotypic assessment

*rgp1* cDNA was isolated from total WT zebrafish cDNA by PCR amplification and purification (28104, Qiagen; Hilden, Germany). The cDNA was inserted into pGEM-T using TA subcloning (A1360, Promega; Madison, WI). Following restriction enzyme digestion, the insert was moved into pCS2+ and linearized by NotI digestion. Synthesized capped mRNA transcripts were produced *via in vitro* transcription with mMESSAGE mMACHINE SP6 transcription kit (AM1340, ThermoFisher Scientific; Waltham, MA). 400 pg of mRNA diluted in 0.3X Danieau Buffer was micro-injected into single cell-stage embryos. Embryos were grown to 4 dpf for subsequent visualization and live imaging. An observer blinded to injection content scored larvae for the extent of the protruding jaw phenotype.

### Generation of transgenic constructs for injection

Transgenic constructs for overexpression of zRgp1, zRab6a, and hRAB8A were generated as previously described using the Gateway cloning-based Tol2kit method (56). Full-length constructs for pME were PCR-amplified with attB1 and attB2 sequences and cloned into pDONR221 using BP clonase II (11789020, ThermoFisher Scientific; Waltham, MA). p3E constructs were PCR-amplified with attB2R and attB3 sites and cloned into pDONR p2R-p3 using BP clonase II. A Multisite Gateway Cloning LR reaction was performed using the p5E-1.7-kb Col2a1a promoter, pME vectors, p3E vectors, pDEST-Tol2pA2, and LR clonase II enzyme mix (11791020, ThermoFisher Scientific; Waltham, MA) (57, 58). The resulting destination clones were used for mosaic overexpression experiments.

Zebrafish embryos at the one-cell stage were injected with 50-100 pg medaka transposase mRNA and 10-100 pg recombined pDEST plasmid. Larvae were collected at 3 dpf for sample processing and imaging.

### Site-directed mutagenesis

Site-directed mutagenesis was performed on plasmids using the Q5 Site-Directed Mutagenesis Kit (E0554S, NEB; Ipswich, MA) as previously described with the following modifications (59). Template DNA was combined with sequence-specific SDM primers generated using NEBaseChanger online software (**Table 1**). Following Q5 PCR amplification, template DNA was degraded, and the newly synthesized product was ligated using the included Kinase/Ligase/DpnI enzyme mix. The final plasmid product was transformed into DH5α E. coli for subsequent plasmid isolation.

**Table 1:** Primers.

Name	Forward	Reverse
sgRNA Oligo	TTTTGCACCGACTCGGTGCCACTTTTTCAAGTTGATAACGGACTAGCCTTATTTTAACTTGCTATTTCTAGCTCTAAAAC	
zrgp1 g1 sequencing	CGTCTCCGGTTGTTTTGTTT	GGTGTGACATTGGGTTTGTG
zrgp1 g3 sequencing	AGGAGCAGTATCAGAGACGACC	ATACTCATGTGACTGGCTTTGTG
zrgp1 cDNA	CGTCTCCGGTTGTTTTGTTT	CGGCACAAGGTAAAACCAGT
zrgp1 coding sequence	ATGATTGAGGTGGTGGCATC	TCAAATATTGATGCTGTTTG
zrgp1-eGFP pCS2+ cloning	CTTAAGATGATTGAGGTGGTGGCATC	CTCGAGCTTGTACAGCTCGTCCATGC
zrgp1 B1-B2	GGGGACAAGTTTGTACAAAAAAGCAGGCTATGATTGAGGTGGTGGC	GGGGACCACTTTGTACAAGAAAGCTGGGTTGCAAATATTGATGCTGTT
zrgp1 B2-B3	GGGGACAGCTTTCTTGTACAAAGTGGCCATGATTGAGGTGGTGGCATC	GGGGACAACTTTGTATAATAAAGTTGCTCAAATATTGATGCTGTTTG
zrgp1 Δ5bp SDM	GGGCCTGTGTTTTTGGCC	CCATAGATGCCACCACCTC
zRab6a B2-B3	GGGGACAGCTTTCTTGTACAAAGTGGTTATGTCTGCAGCAGGAGATTT	GGGGACAACTTTGTATAATAAAGTTGCTTACATTCAGTTCTTTGGCT
hRAB8A B2-B3	GGGGACAGCTTTCTTGTACAAAGTGGCCATGGCGAAGACCTACGA	GGGGACAACTTTGTATAATAAAGTTGCTCACAGTAGCACACAGC
hRAB8A Q67L SDM	ACGGCAGGACtGGAACGATTT	GTCCCATATCTGTAACTTTATCTTC
hRAB8A T22N SDM	GTCGGGAAGAaCTGTGTGCTG	TCCGGAATCCCCGATTAAC
zrgp1 g1 gRNA	AATTAATACGACTCACTATAggtggtggcatctatggcacGTTTTAGAGCTAGAAATAGC	
zrgp1 g3 gRNA	AATTAATACGACTCACTATAggtgtgacgttaagaggcacGTTTTAGAGCTAGAAATAGC	
*edem1* qPCR	ATCCAAAGAAGATCGCATGG	TCTCTCCCTGAAACGCTGAT
*hspa5* qPCR	AAGAGGCCGAAGAGAAGGAC	AGCAGCAGAGCCTCGAAATA
*ddit3p* qPCR	AAGGAAAGTGCAGGAGCTGA	TCACGCTCTCCACAAGAAGA
*ef1* qPCR	GCATACATCAAGAAGATCGGC	GCAGCCTTCTGTGCAGACTTTG
*arf4a* qPCR	CCCATCAGCGAGTTGACAGA	CCCTCGTATAAACCCGTCCC
*arf4b* qPCR	AGAGAATCTCAGCCTCGCAC	ACAGACGCGTCCAAAGGTTA
*tfe3a* qPCR	CGCACGCTGATAGAGGAACT	ACCGATAGCAACCTGTGAGC
*tfe3b* qPCR	AGCCCCATGGCACATCTTAAT	CTCTGGCCTCTGTTTCGATCA
*acbd3* qPCR	AAACCCTGAGCAGAGTGTCG	AGCATCTGGGTTGTAAGGGC

### Cryosectioning and immunohistochemistry

Larvae at 3 dpf were fixed overnight in 4% PFA, washed, and moved to 30% sucrose overnight. The following day, larvae were mounted in OCT embedding medium (6769006, ThermoFisher Scientific; Waltham, MA), frozen, cryosectioned, and transferred onto Superfrost slides (12-550-15, ThermoFisher Scientific; Waltham, MA) as previously described (60). Slides were dried on a heat block, rehydrated in PBS, and mounted onto a Sequenza staining rack. Sections were incubated with Proteinase K for antigen retrieval and permeabilized in PBS+0.5% Triton X-100, then blocked in 2% BSA and 2% NGS. Samples were stained with primary antibodies (**Table 2**) diluted in blocking solution and incubated overnight at 4°C. The following day, samples were rinsed and incubated in secondary antibodies (**Table 2**), rinsed again, and incubated in DAPI before being mounted using Prolong Gold Antifade Agent (P36930, ThermoFisher Scientific; Waltham, MA). Fluorescent imaging was carried out using an AxioImager Z1 equipped with an Apotome and an EC Plan Neofluar 100X/1.30 Oil objective and ORCA-Flash4.0 Digital CMOS Camera. Co-localization analysis of CHP and LBPA was performed using FIJI’s Coloc2 function. Cell boundaries were used to highlight a region of interest, which was then used to make a mask. Pearson’s R values were collected from cells across WT and *rgp1^-/-^* larvae.

**Table 2:** Antibodies/IF Reagents.

Antibody	Catalog No.	Vendor	RRID	Dilution	Species
Collagen II	II-II63B	DSHB	AB_528165	1:250	Mouse IgG1
mCherry	16D7	ThermoFisher Scientific	AB_2536611	1:250	Rat IgG2a
GFP	A10262	Vanderbilt Molecular and Cell Biology Core	AB_2534023	1:500	Chicken IgY
GFP-488	A21311	Invitrogen	AB_221477	1:250	Rabbit IgG
WGA-488	W11261	Invitrogen	N/A	1:500	N/A
CHP-Biotin	B-CHP	3Helix	N/A	1.4µM	N/A
LBPA	Z-PLBPA	Echelon Biosciences	AB_11129226	1:100	Mouse IgG1
Rhodamine-phalloidin	R415	ThermoFisher Scientific	N/A	1:500	N/A
DAPI	D1306	Invitrogen	N/A	1:4000	N/A
Mouse IgG-Alexa647	A32787	Invitrogen	AB_2762830	1:500	Donkey
Rat IgG-Alexa555	A21434	Invitrogen	AB_2535855	1:500	Goat
Mouse IgG-Alexa555	A21422	Life Technologies	AB_2535844	1:500	Goat
Chicken IgY-Alexa488	A11039	Invitrogen	AB_2534096	1:500	Goat
Streptavidin-Alexa555	S32355	Invitrogen	N/A	1:500	N/A

N/A, not applicable.

### Intracellular Col2 quantification

Images of collagen IF were analyzed in FIJI. For collagen accumulation in chondrocytes, the intracellular area of each cell (demarcated by caax-eGFP signal), collagen area (Col2 signal), and nuclear area (DAPI) were measured. The following formula was used to calculate the percent intracellular collagen area per cell (18):


$$\%\;intracellular\;collagen\;II=\frac{collagen\;II\;area}{\left(intracellular\;area-nuclear\;area\right)}\times 100$$

### Electron microscopy

Larvae were fixed at 3 dpf in 2.5% glutaraldehyde in 0.1 M sodium cacodylate by incubating at room temperature for 1 hour first, then overnight at 4°C, as previously described (18). After rinsing in 0.1 M sodium cacodylate, samples were post-fixed with 1% osmium tetroxide in 0.1 M sodium cacodylate for 1 hour. After additional rinsing, specimens were sequentially dehydrated in increasing concentrations of ethanol and then propylene oxide, infiltrated with resin step-wise, and then embedded in resin for 48 hours at 60°C. Coronal sections of 10 µm were collected until craniofacial chondrocytes were visible under low magnification microscopy based on their location in the jaw, characteristic stacking arrangement, and surrounding ECM. 70-nm sections were collected on TEM grids following sectioning on a Leica Ultracut Microtome and analyzed on a Phillips CM-12 Transmission Electron Microscope provided by the Vanderbilt Cell Imaging Shared Resource. Images of the hyosymplectic element were acquired from tiled images and stitching across single sections. Individual ultrastructural elements were identified by morphological characteristics and were pseudo colored.

### Live vesicle movement and tracking

Live vesicle movement in WT and *rgp1^-/-^* transgenic larvae was performed on a Zeiss LSM880 confocal microscope. Embryos obtained from gRNA-generated stable mutant lines crossed to mosaic (Col2a1a:caax-eGFP) adults were injected with a Tol2 construct expressing Col2a1a:mCherry-zRab6a for transient, transposon-mediated DNA integration. Zebrafish larvae at 3 dpf that were grown in PTU were selected for imaging if they expressed caax-eGFP and expressed substantial mCherry-zRab6a in the hyosymplectic cartilage. Larvae were anesthetized in 0.15 mg/mL Tricaine, embedded in 1.2% low-melt agarose (A20070, RPI; Mount Prospect, IL) on glass-bottom confocal dishes, and overlaid with embryo media containing 0.4 mg/mL Tricaine. For time-lapse imaging, larvae were imaged under a 40X/1.1 LD C-Apochromat water-immersion objective. Frame intervals of 300 ms were collected over the course of 300 frames for each sample with 300 ms delay between acquisitions. Individual frames were deconvolved using Airyscan (Zeiss; Jena, Germany). **Supplemental Movies 1**, **2** are sped up to 100 frames/second for display purposes.

Confocal time-lapse images from live transgenic larvae were analyzed in Imaris 9.7.2 (Oxford Instruments; Abingdon, United Kingdom). Analysis was performed using the automated Spots feature. Spots in the red channel were filtered by their quality and fit to the autoregressive motion-tracking algorithm. Tracks generated over longer than 5 frames were selected for analysis. All tracks collected were measured for track length and mean track speed.

### TUNEL assay

To identify double-stranded DNA breaks indicative of cell death, TUNEL assays were performed as previously described with the following modifications (61). Larvae were fixed overnight in 4% PFA, moved to 30% sucrose in PBS, and cryosectioned. TUNEL was performed using the Roche *In Situ* Death Detection Kit (12156792910, Sigma; St. Louis, MO). Larvae were co-stained with WGA-488 or GFP-488 and DAPI.

### qPCR

Synthesized cDNA was diluted 1:1 with water before use in qPCR. Three biological replicates were performed, with each biological replicate consisting of an independent RNA extraction. qPCR was performed using forward and reverse primers (**Table 1**), cDNA, and 2X SYBR Green PCR Master Mix (4309155, ThermoFisher Scientific; Waltham, MA) as previously described (62). Samples were run in technical triplicates, and curves were normalized to *ef-1* transcript levels.

### Statistical analysis

Statistical analyses were performed with GraphPad Prism 9.3.1 (63). Statistical significance between WT and *rgp1^-/-^* larva gross morphology was assessed by Mann-Whitney U-test (two-tailed) with 95% confidence intervals. For head length and cartilage size measurements, symbols on the graph represent individual larvae, with lines representing means ± SEM.

Statistical significance for jaw protrusion phenotypes following mRNA injection was analyzed by Chi-Square analysis. Numbers on the graph represent larvae across pooled clutches from injections across at least three independent mating crosses.

Statistical significance between mCherry-zRab6a puncta dynamics in WT and *rgp1^-/-^* chondrocytes was assessed by Mann-Whitney U-test (two-tailed) with 95% confidence intervals. Symbols on graph represent individual vesicles with red lines representing means.

Comparisons of intracellular collagen II staining between fluorescent and non-fluorescent chondrocytes was determined by one-way ANOVA with Tukey’s multiple comparison test. Symbols on the graph represent measurements of individual chondrocytes with lines representing means ± SEM.

Statistical analysis of cell death phenotypes in TEM of chondrocytes was not performed. Numbers on the graph represent the fraction of total individual chondrocytes with each phenotype.

Statistical analysis between qPCR expression levels was assessed by Mann-Whitney U-test (two-tailed) with 95% confidence intervals. Symbols on the graph represent individual biological replicates from technical triplicates with lines representing means ± SEM.

No data points or measurements were omitted across any experiment. The number of larvae, vesicles, or cells required for each experiment was determined based on power analysis with 95% confidence intervals. Significance is represented by *p<0.05; **p<0.01; ***p<0.001; ****p<0.0001; ns, not significant.

## Results

### CRISPR-generated *rgp1^-/-^* zebrafish larvae have craniofacial defects

Zebrafish begin to develop a protruding jaw around 3 days post-fertilization (dpf), a process essential for feeding that begins at 5 dpf. Previous work from our laboratory noted that mosaically reduced Rgp1 expression contributes to defects in jaw morphology and craniofacial development (18). However, it remains unknown how genetic mutations in Rgp1 affect craniofacial cartilage development. To generate stable mutant lines, we used CRISPR/Cas9 genome editing and two guide RNAs (gRNAs) targeting exons one (g1) and seven (g3) to generate three independent *rgp1* mutant alleles (**Figures 1A, B**). All three zebrafish alleles are predicted to induce frameshift mutations, which would likely result in the translation of a shorter peptide or nonsense mediated decay (**Figure 1C**). All three *rgp1^-/-^* alleles showed a lack of a protruding jaw and were shorter when compared to wild-type (WT) controls (**Figure 1D**). The embryonic zebrafish craniofacial skeleton is primarily cartilaginous until 5 dpf (64). To assess the cartilage structure and integrity, we stained larvae with Alcian blue, a dye that binds glycosaminoglycans commonly found in the cartilage extracellular matrix (**Figure 1E**) (54). In *rgp1^-/-^* larvae, we found malformed craniofacial cartilage elements, leading to an overall shortening of the head skeleton by approximately 20% compared to WT controls (**Figure 1F**, p<0.0001). The Meckel’s cartilage, corresponding to the lower jaw, is significantly shorter in *rgp1^-/-^* larvae (**Figures 1E, G**, p<0.0001). Additionally, the ceratohyal cartilage element, a ventral structure of the head skeleton, was bent at a wider angle in mutants compared to WT controls (**Figures 1E, H**, p<0.0001).

To validate our new model and rule out the possibility of CRISPR-induced off-target effects, we performed an mRNA genetic replacement experiment and assessed jaw morphology in live larvae to determine if clutches overexpressing *rgp1* had a majority of larvae with normal protruding jaws (**Figures 1I–J**) (65–67). There was an increase in the proportion of larvae showing a protruding jaw in WT mRNA injected larvae, compared to the non-injected control of 75% larvae with protruding jaws, typical for a recessive phenotype (**Figure 1K**, p<0.0001). On the contrary, injection of an mRNA coding for the mutant *rgp1^g1 -5^* allele did not result in phenotypic jaw rescue (**Figure 1K**, p=0.502). The *rgp1^g1 -5^* allele was used in panels D-K, although all three alleles presented with a similar phenotype. Similarly, trans-heterozygote larvae for different *rgp1* mutant alleles had similar jaw protrusion defects to homozygous mutant alleles, further ruling out the possibility of off-target effects in individual *rgp1* mutant lines (data not shown). From the mRNA genetic replacement experiment, we concluded that the observed craniofacial skeletal defects were due to mutations in *rgp1* and its subsequent loss of function.

### Rab6a activation is reduced in *rgp1^-/-^* chondrocytes

**Figure 2:**
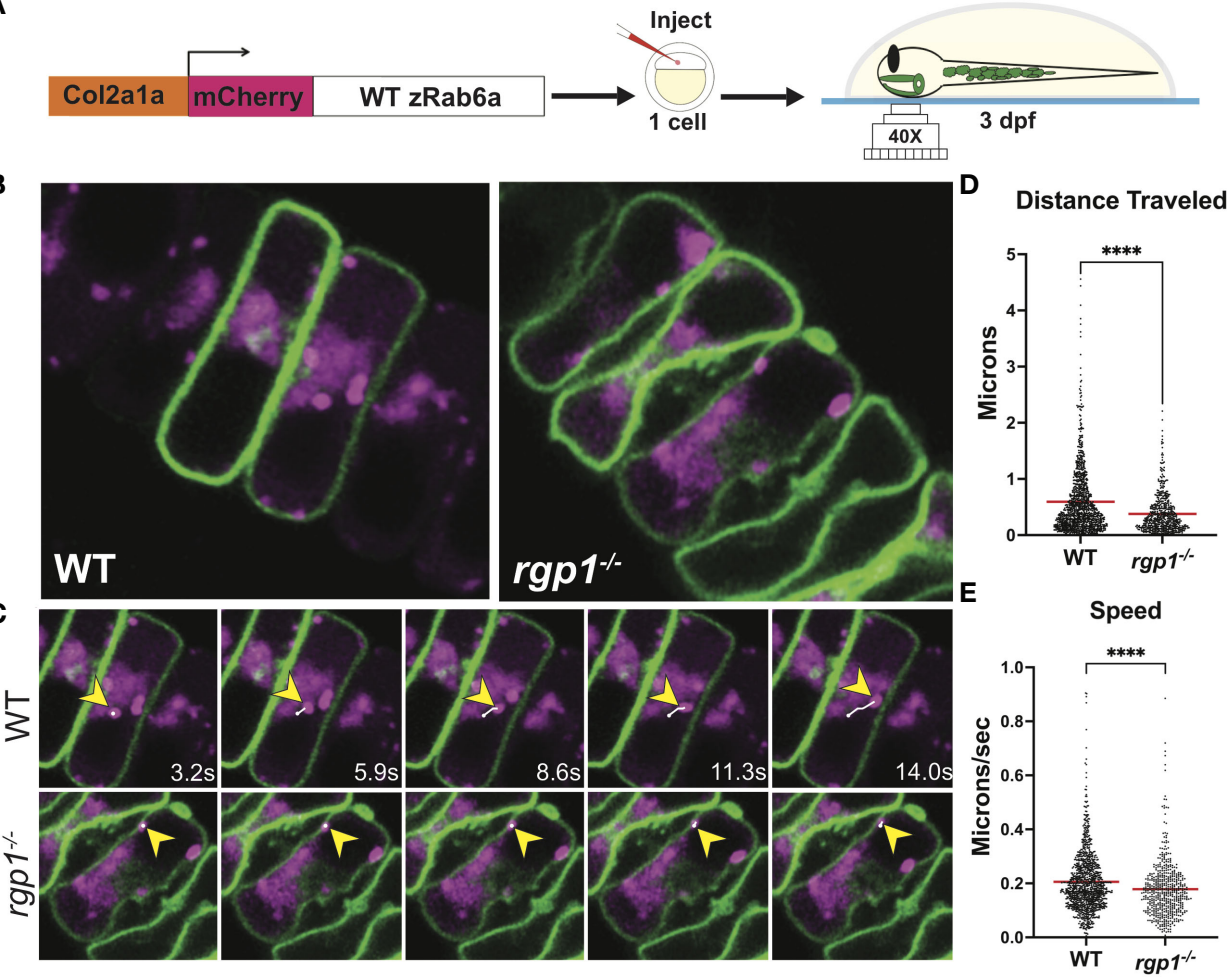
Rab6a vesicle trafficking is reduced in *rgp1^-/-^* chondrocytes **(A)** Experimental design for mosaic overexpression of WT mCherry-zRab6a fusion protein in Tg(Col2a1α:caax-eGFP) transgenic zebrafish chondrocytes for live imaging. **(B)** Representative images from movies of mCherry-zRab6a vesicular compartment movement in chondrocytes (**Supplemental Movies 1, 2**). **(C)** Montage of mCherry-zRab6a puncta progression at specific times in WT and *rgp1^-/-^* chondrocytes (yellow arrowheads) showing path of indicated vesicle over time (white line). **(D)** Quantification of vesicular compartment distances traveled in WT and *rgp1^-/-^* chondrocytes. Red lines indicate means. **(E)** Quantification of vesicular compartment speeds in WT and *rgp1^-/-^* chondrocytes. Red lines indicate means. Significance is presented by ****p<0.0001.

Rgp1 was previously identified as a component of the Rab6a GEF complex in yeast and mammalian cells (44, 45). To test whether mutations in *rgp1* disrupt Rab6a activation and vesicle movement in our zebrafish model, we generated and injected a Tol2 DNA construct to overexpress mCherry-fused WT zebrafish Rab6a (WT zRab6a) under a Col2a1α promoter (57, 58, 68). We co-injected the construct along with transposase into the single-cell progeny of heterozygote mutant fish carrying a transgene that labels chondrocyte membrane with caax-eGFP (**Figure 2A**) (69). Because transposase integration is mosaic, not all cells express the construct, and the mCherry fusion protein highlights specific vesicular compartments. This gave us the opportunity to compare activation of Rab6a and vesicular compartment movements as puncta in movies. We chose to analyze hyosymplectic chondrocytes that stack horizontally, develop around 3 dpf, and are close to the zebrafish skin, making them amenable to *in situ* live imaging (**Figures 2B, C**) (64). We found that the mCherry-zRab6a^+^ vesicular compartments in *rgp1* mutants traveled shorter distances (**Figure 2D**, p<0.0001) and moved slower (**Figure 2E**, p<0.0001) than WT controls (**Supplemental Movies 1, 2**). These data suggest that Rgp1 is needed for Rab6a^+^ vesicular compartment dynamics within chondrocytes.

### Rgp1 is required for collagen II secretion

**Figure 3:**
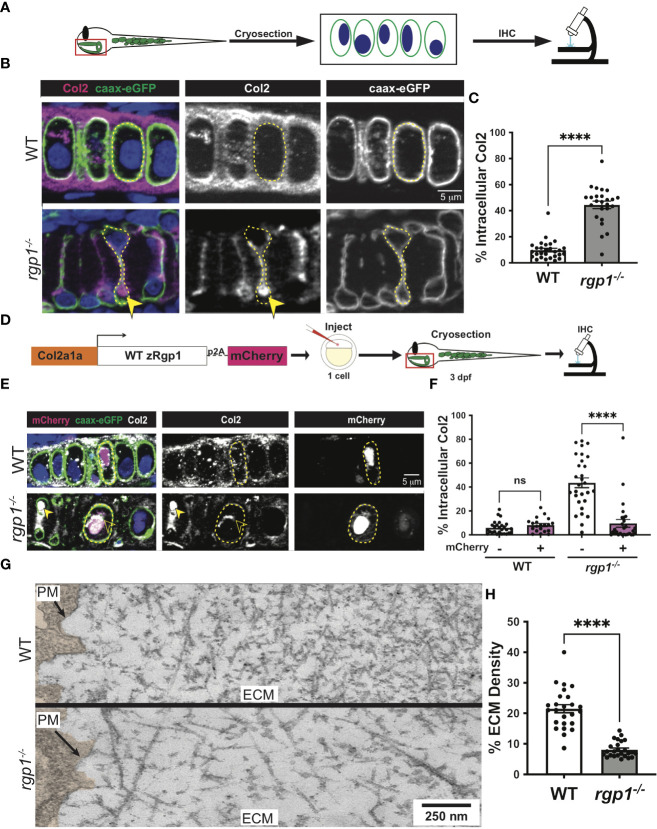
*Rgp1^-/-^* chondrocytes accumulate collagen II intracellularly. **(A)** Experimental design for immunohistochemical analysis of chondrocytes in the craniofacial head skeleton. **(B)** Representative images of Tg(Col2a1α:caax-eGFP) transgenic larvae in WT and *rgp1^-/-^* cartilage (Col2, magenta; eGFP, green). Yellow arrowhead identifies intracellular collagen II. **(C)** Quantification of the percentage of cytosolic area occupied by collagen II signal in chondrocytes. **(D)** Experimental design for mosaic overexpression of zRgp1-p2A-mCherry in zebrafish chondrocytes. **(E)** Representative images of Tg(Col2a1α:caax-eGFP) transgenic chondrocytes in WT and *rgp1^-/-^* cartilage (TEM images of chondrocyte membranes (PM) and adjacent mCherry, magenta; eGFP, green; Col2, white). Yellow arrowhead identifies collagen II in mCherry^-^ cells while empty arrowhead identifies collagen II in mCherry^+^ cells. **(F)** Quantification of the percentage of cytosolic area occupied by collagen II signal in chondrocytes. **(G)** TEM images of chondrocyte membranes and adjacent ECM proteins. **(H)** Quantification of TEM staining density from craniofacial chondrocyte plasma membrane to edge of cartilage tissue using ImageJ. Significance is presented by ****p<0.0001; ns, not signficant.

Craniofacial chondrocytes predominantly secrete collagen II during larval craniofacial development (68). To assess secretion of collagen II, we performed immunofluorescence with a collagen II antibody in WT and *rgp1^-/-^* larvae crossed to a transgenic zebrafish line expressing membrane-bound caax-eGFP under the Col2a1α promoter (**Figure 3A**) (57, 69, 70). The caax-eGFP transgene labeled the cell membrane boundary to distinguish intracellular from extracellular space. We identified two prominent phenotypes in the *rgp1^-/-^* chondrocytes: collagen II secretion defects and an abnormal cell shape; we have chosen to focus on the collagen II secretion phenotype. We found that *rgp1^-/-^* chondrocytes had significantly higher amounts of intracellular collagen II when compared to WT controls (**Figures 3B, C**, p<0.0001). We also observed reduced extracellular collagen II staining in *rgp1^-/-^* cartilage. Thus, we conclude that Rgp1 is required for secretion of collagen II in chondrocytes.

To test whether the intracellular collagen II accumulations stem specifically from loss of Rgp1, we overexpressed WT zebrafish Rgp1 (zRgp1) under a Col2a1α promoter using a Tol2 construct (**Figure 3D**). We took advantage of a viral p2A self-cleavable peptide sequence to identify cells over-expressing WT zRgp1 (56, 71). Single-cell embryos were injected, and the construct mosaically integrated, with cytosolic mCherry expression only visible in cells where the Tol2 construct integrated (**Figure 3E**). WT chondrocytes had no significant difference in the amount of intracellular collagen II regardless of whether they overexpressed zRgp1 or not (**Figure 3F**, p=0.960). In *rgp1*-deficient chondrocytes, expression of zRgp1-p2A-mCherry (**Figure 3E**) reduced accumulations of intracellular collagen II compared to mCherry^-^ chondrocytes (**Figures 3E, F**, p<0.0001). Thus, overexpression of WT zRgp1 in *rgp1^-/-^* chondrocytes was sufficient to rescue collagen II secretion defects in a cell-autonomous manner.

To assess the abundance of extracellular matrix proteins in an antibody-independent method, we used TEM. We found that at 3 dpf, WT chondrocytes have secreted a large amount of protein to the ECM, with increasing crosslinking further away from the cell membrane (**Figure 3G**). However, *rgp1*-deficient cartilage ECM was significantly less dense compared to WT (**Figure 3H**, p<0.0001), although there were ECM proteins present in the extracellular space. This supports our observation that although collagen II is likely not secreted by *rgp1^-/-^* chondrocytes, other ECM proteins are secreted to the extracellular space.

### Collagen II accumulates in an endolysosomal compartment

**Figure 4:**
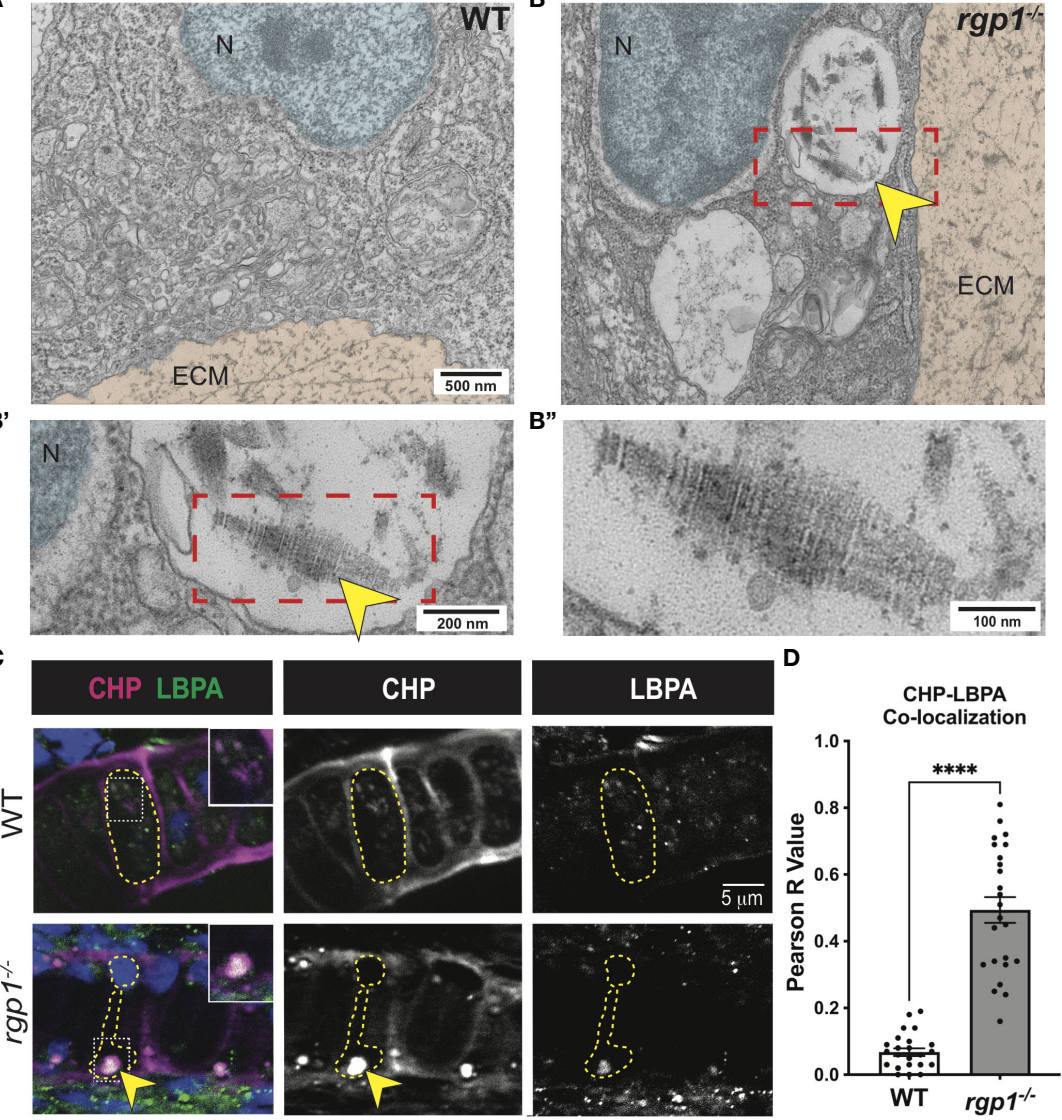
Collagen II accumulations in *rgp1^-/-^* chondrocytes localize to endolysosomes **(A)** TEM images of 3 dpf WT craniofacial chondrocytes. **(B)** TEM images of 3 dpf *rgp1^-/-^* craniofacial chondrocytes showing vacuolar structures (yellow arrowhead) **(B’)** Enlarged image of boxed area showing striated ultrastructure of intracellular collagen fibrils (yellow arrowhead). **(B”)** Further enlargement of boxed area. **(C)** Representative images of chondrocytes in WT and *rgp1^-/-^* cartilage (CHP, magenta; LBPA, green). Insets are enlargements of white dashed boxes, and an intracellular accumulation is labeled with a yellow arrowhead. **(D)** Quantification of the Pearson R value for co-localization in individual chondrocytes. Significance is presented by ****p<0.0001.

To assess where collagen II accumulates in *rgp1^-/-^* chondrocytes, we compared TEM images of WT and *rgp1^-/-^* hyosymplectic chondrocytes (**Figure 4A**). In *rgp1^-/-^* cells, we found large vacuolar-like structures, (**Figure 4B**) some of which were filled with striated assemblies (**Figure 4B’-B”**). Previous studies have identified this striated TEM ultrastructure as a fully assembled, mature collagen fibril, which is typically found exclusively extracellularly (72, 73). Collagen II is normally trafficked with N- and C-terminal telopeptide domains that prevent aberrant oligomerization intracellularly (4, 74, 75). The presence of striated collagen intracellularly suggests that endopeptidases cleaved the procollagen trimer, thus allowing for spontaneous fibril assembly within the observed vacuolar-like structures.

Lysosomes are small, vacuolar compartments that concentrate proteolytic peptidases for enzymatic digestion (76). To test whether the structures observed in TEM carry hallmarks of lysosomal membranes containing collagen, we co-stained for markers of endolysosomal compartments and collagen. To perform this co-localization assay, we used an antibody against a lipid enriched in endolysosomal membranes, lysobisphosphatidic acid (LBPA), and a collagen hybridizing peptide (CHP; **Figure 4C**) (77, 78). We chose to use CHP instead of a collagen II antibody since the collagen II antibody and the LBPA antibody were generated in the same host species. In WT chondrocytes, we found LBPA staining in small puncta, consistent with staining of endolysosomal compartments; we also found very little co-localization between LBPA and CHP, suggesting that collagen is not normally found in LBPA^+^ compartments (**Figure 4C**). However, CHP and LBPA staining often co-localized in *rgp1^-/-^* chondrocytes (**Figures 4C, D**, p<0.0001) and was as large as the inclusions in the TEM images. This suggests that collagen II accumulates in an endolysosomal compartment in *rgp1^-/-^* chondrocytes.

### Rab8a is required for collagen II trafficking, and constitutive activation of Rab8a partially rescues *rgp1^-/-^* collagen II secretion defects

**Figure 5:**
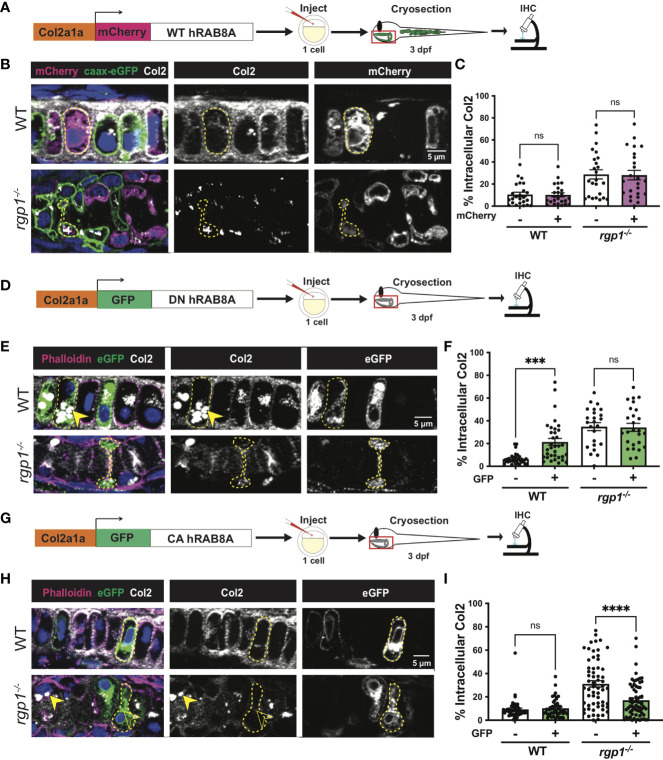
Collagen II trafficking is regulated by Rab8a. **(A)** Experimental design for mosaic overexpression of WT mCherry-hRAB8A fusion protein in zebrafish chondrocytes. **(B)** Representative images of Tg(Col2a1α:caax-eGFP) transgenic chondrocytes in WT and *rgp1^-/-^* cartilage (mCherry, magenta; caax-eGFP, green; Col2, white). **(C)** Quantification of the percentage of cytosolic area occupied by collagen II signal in chondrocytes. **(D)** Experimental design for mosaic overexpression of DN GFP-hRAB8A fusion protein in zebrafish chondrocytes. **(E)** Representative images of chondrocytes in WT and *rgp1^-/-^* cartilage (phalloidin, magenta; eGFP, green; Col2, white). Yellow arrowhead identifies intracellular collagen II. **(F)** Quantification of the percentage of cytosolic area occupied by collagen II signal in chondrocytes. **(G)** Experimental design for mosaic overexpression of CA GFP-hRAB8A fusion protein in zebrafish chondrocytes. **(H)** Representative images of chondrocytes in WT and *rgp1^-/-^* cartilage (phalloidin, magenta; eGFP, green; Col2, white). Yellow arrowhead identifies collagen II in GFP^-^ cells while empty arrowhead identifies collagen II in GFP^+^ cells. **(I)** Quantification of the percentage of cytosolic area occupied by collagen II signal in chondrocytes. Significance is presented by ***p<0.001; ****p<0.0001; ns, not signficant.

Rab8a has been shown to mainly associate with exocytic trafficking from the *trans*-Golgi network (TGN) to the plasma membrane and co-localizes with Rab6a^+^ vesicular carriers (79–83). To test whether overexpression of hRAB8A is sufficient to affect collagen II secretion in zebrafish chondrocytes, we used WT mCherry-hRAB8A under a Col2a1α promoter (**Figure 5A**). In WT larvae, there was no difference in intracellular collagen II levels between mCherry^+^ and mCherry^-^ cells (**Figure 5B, C**, p=0.9999). In *rgp1^-/-^* chondrocytes, we found that overexpression of the WT mCherry-hRAB8A also had no effect on intracellular collagen II when compared to mCherry^-^ cells (**Figures 5B**, **5C**, p=0.9993). Thus, simple overexpression of WT hRAB8A does not alter collagen II secretion.

Point mutations in Rab proteins can alter their affinity for GTP or GDP to lock it in the “constitutively-active” (CA), GTP-bound conformation or the “dominant-negative” (DN), GDP-bound conformation (48, 84–86). To test whether inactivating Rab8a blocks collagen II secretion in zebrafish chondrocytes, we expressed a Tol2 fusion construct of GFP and DN human RAB8A (T22N) under a Col2a1α promoter (**Figure 5D**) (85). We found that GFP^+^ WT chondrocytes accumulated large inclusions of collagen II in higher amounts compared to their adjacent GFP^-^ counterparts (**Figures 5E, F**, p=0.0004). In *rgp1^-/-^* chondrocytes that already accumulate large collagen II inclusions, there was no change in intracellular collagen II staining in GFP^+^ vs GFP^-^ cells (**Figure 5F**, p>0.9999). This result is consistent with Rab8a being required for cargo secretion, including collagen II in chondrocytes.

To test whether constitutive activation of hRAB8A would be sufficient to rescue collagen II accumulations in *rgp1^-/-^* chondrocytes, we expressed a Tol2 fusion construct of GFP and CA hRAB8A (Q67L) under a Col2a1α promoter (**Figure 5G**) (86). In WT chondrocytes, there was no difference in intracellular collagen II levels between GFP^+^ and GFP^-^ cells (**Figures 5H, I**, p=0.9890). In *rgp1^-/-^* chondrocytes, we found that expression of CA GFP-hRAB8A (**Figure 5H**) reduced levels of intracellular collagen II compared to GFP^-^ cells (**Figures 5H, I**, p<0.0001). Therefore, overexpression of CA Rab8a is sufficient to partially rescue secretion of intracellular collagen II in *rgp1^-/-^* chondrocytes, suggesting that Rgp1 functions upstream of the Rab8a activation step.

### *Rgp1* ^-/-^ chondrocytes activate Golgi stress and undergo cell death

**Figure 6:**
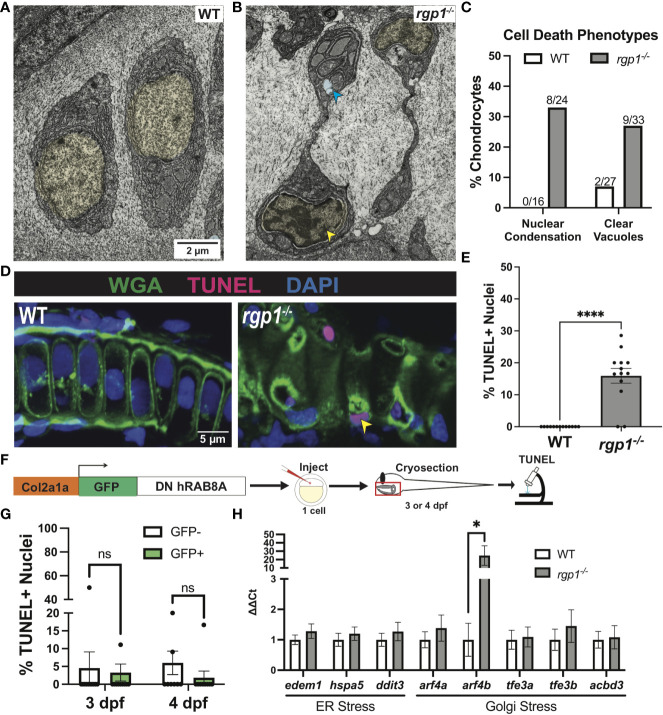
*rgp1^-/-^* chondrocytes undergo cell death and induce a Golgi stress marker. **(A)** TEM images of 3 dpf WT chondrocytes showing normal cellular morphology. **(B)** *rgp1^-/-^* craniofacial chondrocytes showing partial nuclear condensation (yellow shading, blue arrow) and clear cytoplasmic vacuoles (blue shading, blue arrow). **(C)** Quantification of cell death-associated TEM phenotypes. **(D)** Representative images of chondrocytes labeled with TUNEL and WGA in WT and *rgp1^-/-^* cartilage (TUNEL, magenta; WGA, green). Yellow arrowhead identifies TUNEL^+^ nucleus. **(E)** Quantification of the percentage of nuclei that were TUNEL^+^ in each image. **(F)** Experimental design for mosaic overexpression of DN GFP-hRAB8A fusion protein in zebrafish chondrocytes, as used in **Figure 5A**. **(G)** Quantification of the percentage of nuclei that were TUNEL^+^ in each image at 3 dpf or 4 dpf **(H)** qPCR panel of ER stress transcripts (*edem1*, *bip*, *chop*) and Golgi stress transcripts (*arf4a, arf4b, tfe3a, tfe3b, acbd3*) normalized to *ef-1* transcript levels in 3 dpf larvae. Significance is presented by *p<0.05; ****p<0.0001; ns, not signficant.

In TEM analysis at 3 dpf, we observed *rgp1^-/-^* chondrocytes with abnormal nuclei when compared to WT cells (**Figures 4A, B**). The observed mixture of electron-dense and electron-sparse material within the nucleus is suggestive of nuclear condensations, an early sign of cell death. To identify hallmarks of cell death, we further assessed chondrocyte ultrastructure using TEM on hyosymplectic chondrocytes (87, 88). While WT chondrocytes did not have any abnormalities in nuclear structure, *rgp1^-/-^* chondrocytes had begun to undergo nuclear condensation and presented with clear cytoplasmic vacuolar structures (**Figures 6A, B**). Specifically, binary scoring for the presence or absence of these phenotypes revealed that hallmarks of cell death were only sporadically observed in the WT chondrocytes (**Figure 6C**). As such, these findings suggest that *rgp1^-/-^* chondrocytes undergo cell death at higher rates than WT chondrocytes.

To test if *rgp1^-/-^* chondrocytes underwent DNA fragmentation consistent with the nuclear condensation observed in TEM, we performed a TUNEL assay. As a co-stain, we used wheat germ agglutinin (WGA) to label N-glycosylated proteins in the extracellular matrix and to help detect cell boundaries (**Figure 6D**). We found more TUNEL^+^ nuclei in *rgp1^-/-^* chondrocytes compared to WT nuclei, indicative of higher levels of DNA fragmentation (**Figure 6E**, p<0.0001). Together, the nuclear changes detected by TEM and TUNEL staining support the increase in chondrocyte cell death in Rgp1-deficient cells.

It remained unclear whether the observed cell death occurred as a result of collagen II accumulations or if another Rgp1-related phenotype induced cell death. To test whether collagen II accumulation induced cell death, we overexpressed DN GFP-hRAB8A (**Figure 5D**) in WT chondrocytes and compared TUNEL staining in GFP^+^ cells with intracellular collagen II accumulations to GFP^-^ chondrocytes with no collagen II backlogs (**Figure 6F**). We found no difference in the percentage of nuclei with DNA fragmentation between the GFP^+^ and GFP^-^ chondrocytes at 3 dpf (**Figure 6G**, p>0.9999); this observation was consistent even after prolonged accumulation of collagen II at 4 dpf (**Figure 6G**, p=0.3294). Thus, we conclude that the chondrocyte cell death in *rgp1^-/-^* chondrocytes is caused independently from collagen II accumulation.

Since collagen II accumulation was not sufficient to induce cell death, we investigated whether defects in Golgi trafficking could cause cellular stress responses along the secretory pathway. To test this possibility, we used qPCR to measure transcript levels of three ER stress transcripts, *edem1*, *hspa5* (BiP), and *ddit3* (CHOP), and five Golgi stress transcripts, *arf4a, arf4b, tfe3a, tfe3b, and acbd3* in 3 dpf larvae (**Figure 6H**). However, *rgp1^-/-^* larvae significantly upregulated expression of *arf4b* (**Figure 6H**, p=0.0286), an ortholog of human ARF4 (GTPase of the Ras superfamily of small G proteins), that was previously shown to be upregulated in response to Golgi stress conditions impinging on Golgi integrity and homeostasis (89). Upregulation of Arf4b and the fact that TEM imaging of *rgp1^-/-^* chondrocytes failed to identify canonical Golgi stacks (data not shown) similar to those observed in WT siblings, suggests that *rgp1* loss-of-function leads to Golgi stress and Golgi fragmentation.

## Discussion

While studies of trafficking mechanisms *in vitro* and in yeast have been informative, studies in multicellular organisms are critical to identify the trafficking mechanisms required for tissue organization. This is especially true in vertebrates where the coordinated secretion of large cargoes is essential for normal development. Prior work in the field recognized that the TGN serves as a sorting hub for exocytic vesicles shown to be heterogeneous in their cargoes, trafficking regulators, and destinations (90). Here, we show that Rgp1 is required for the post-Golgi secretion of select protein cargoes. As a result, Rgp1-deficient zebrafish larvae present with prominent craniofacial cartilage phenotypes including (i) disrupted collagen II trafficking and protein backlogs within chondrocytes, (ii) activation of a Golgi stress response and increased chondrocyte death, and (iii) altered chondrocyte cell shape.

Studies in yeast and mammalian cells in culture identified Rgp1 as part of a GEF complex for Rab6a (44, 45). In our study, we show that Rgp1 is essential for Rab6a activation *in vivo* in zebrafish chondrocytes (**Figure 2**). Consistent with a conserved mechanism of Rgp1 function among vertebrates from zebrafish to humans, our *in vivo rgp1^-/-^* model revealed an essential role for Rgp1 in craniofacial development and collagen II secretion.

Our results revealed that in chondrocytes, Rgp1 is required for the trafficking of a subset of ECM cargoes from the TGN. Using TEM imaging of *rgp1^-/-^* cartilage, we found a limited amount of protein in the extracellular space (**Figure 3**). Additionally, immunofluorescence experiments revealed a small amount of collagen II present in the *rgp1^-/-^* cartilage extracellular space. These findings suggest that collagen II secretion is inefficient following reduced Rab6a pathway activation or there exists a potential compensatory mechanism for collagen II secretion. Previous work in the field has shown that the ER contacts the plasma membrane at distances sufficient for direct trafficking (91–93). It is plausible that in *rgp1^-/-^* cartilage, a small amount of collagen II is secreted directly from the ER to the extracellular space as an adaptive mechanism during Golgi stress. Alternatively, some collagen II in the Golgi could be packaged into exocytic vesicles regulated by other proteins, like Arf GTPases (40, 94). Arf proteins, like Rabs, regulate trafficking from the *trans*-Golgi to different compartments within the cell, including the plasma membrane (95).

Previous findings from mammalian cell culture experiments show that Rab6a coordinates vesicle trafficking through a post-Golgi Rab cascade together with Rab8a (80, 96). By overexpressing DN hRAB8A, we showed that Rab8a is required to traffic collagen II in zebrafish chondrocytes. Overexpression of CA hRAB8A rescued collagen II accumulations in *rgp1^-/-^* chondrocytes, suggesting that Rab8a acts downstream of Rgp1 within the secretory pathway. It has been appreciated that Rab8a regulates post-Golgi trafficking of small cargoes such as the transferrin receptor, vesicular stomatitis virus G protein, and matrix metalloproteinase 14 (81, 86, 97). Rab8a is also required in polarized cells to traffic collagen IV, a network collagen important for basement membrane organization (98). Here, we provide evidence that in non-polarized chondrocytes, Rab8a regulates the trafficking of collagen II, a large fibrillar collagen in cartilage ECM. This suggests that in other tissues, Rab8a might also be involved in the traffic of diverse large cargoes, expanding the essentiality of Rab8a during development.

We found that in Rgp1-deficient chondrocytes, type II collagen accumulates in the endolysosomal compartment. Potentially, collagen II arrives at the late Golgi but is unable to continue along the secretory pathway because of the dysfunctional TGN. Stalled membrane bound compartments are likely recognized by a stress response mechanism (e.g. Arf4), leading to fusion with lysosomes to clear intracellular accumulations. Our TEM findings confirmed the presence of a large number of small, membrane-bound compartments loaded with electron dense material and large vacuolar-like structures containing processed and assembled collagen fibers. TEM images showing reduced cartilage ECM density further correlate the two findings. Furthermore, DN hRAB8A overexpression to block collagen II secretion was not sufficient to induce cell death, suggesting that another Rgp1-mediated process causes cell death.

Our identification of an Arf4b-specific Golgi stress response aligns well with a previous study in human cells in culture that found Arf4 upregulation following application of Golgi-disrupting drugs (89). While intriguing, that study used drugs known to induce both Golgi stress and ER stress (99, 100). In contrast, our model identifies a Golgi-specific stress response in the absence of an ER stress response. Arf4 has been implicated in regulating transport between the recycling endosome and TGN, and increased *arf4* expression levels were shown to correlate with Golgi fragmentation (89, 101). Thus, it is not surprising that in the absence of Rgp1 activity, leading to a dysfunctional TGN and buildup of matrix proteins in the endolysosomal compartment, *arf4* becomes upregulated.

Extensive analyses of TEM images from *rgp1^-/-^* chondrocytes failed to identify canonical Golgi stacks similar to those observed in WT counterparts. It is conceivable that disturbed Rab6a-mediated traffic contributes to Golgi disorganization or even dispersion (102). Since Rgp1 GEF activity is associated with the TGN, it is feasible that Rgp1 deficiency could lead to Golgi dispersion and stress, despite a normally functioning ER (44). This is consistent with our live imaging data tracking Rab6a^+^ compartments, where WT chondrocytes showed perinuclear and condensed structures, while *rgp1^-/-^* chondrocytes showed more dispersed structures. Future studies will be needed to understand the relationship of Golgi morphology and Golgi stress.

Our study also poses new questions regarding mechanisms regulating cargo trafficking and developmental cell shape changes in mesenchymal cells such as chondrocytes. This highlights the existence of alternative pathways as compared to those identified in polarized, basement membrane oriented epithelial cells. Chondrocyte shape variation was observed in cells deficient in various components of the secretory pathway machinery including COPII (Sec23, Sec24) or post-Golgi elements (Ric1, Kif5b, Erc1) (9, 10, 18, 23, 103). Unlike genetic replacement of Rgp1 in *rgp1^-/-^* larvae that rescued all cellular phenotypes, overexpression of constitutively activated hRAB8A was not able to overcome the *rgp1^-/-^* cell shape phenotype, suggesting that increased exocytic pathway activation is not sufficient to restore cell shape (**Figure 5**). This suggests that there are yet unknown processes in cell shape determination regulated by Rgp1.

Despite a growing body of knowledge in the field of cartilage and bone biology, surprisingly few diseases have been described involving the secretory machinery, particularly in the post-Golgi compartment (8). Recently, we have described the novel CATIFA syndrome, where subjects carry variants in RIC1, the binding partner of RGP1 (18). Deficiencies of RIC1 in CATIFA and Rgp1 in zebrafish models are consistent with type II collagenopathies, affecting collagen II secretion, and their common presentation includes scoliosis, cleft palate and a shorter jaw (31, 104). Although, no syndromes have been currently linked to the *RGP1* gene, the zebrafish models presented here will provide a valuable tool to test other non-skeletal phenotypes potentially associated with RGP1 variants that might emerge from Whole Exome Sequencing of clinical cases.

In closing, regulation of post-Golgi trafficking is an essential process in the development and function of tissues such as cartilage. The coordination of Rgp1, Rab6a, and Rab8a to regulate collagen II secretion demonstrates the complexity of intracellular interactions that govern vesicle dynamics. The continued identification of the individual components and pathways that regulate protein trafficking offers insight into biological mechanisms that are likely to apply broadly across tissues during development.

## Data availability statement

The data presented in this study are deposited in Genbank (accession numbers OQ305607, OQ305608, OQ305609, OQ305610) and Figshare (accession numbers 21944015, 21944093).

## Ethics statement

The animal study was reviewed and approved by Institutional Animal Care and Use Committee at Vanderbilt University Medical Center.

## Author contributions

DR, GU, and EK conceived the project and designed the analysis. DR and DC collected the data. DR, DC, and GU contributed analysis tools. DR performed most of the research and analysis described. DR and EK wrote the paper with contributions and input from all authors. All authors contributed to the article and approved the submitted version.
